# In-Plane Behaviour of a Reinforcement Concrete Frame with a Dry Stack Masonry Panel

**DOI:** 10.3390/ma9020108

**Published:** 2016-02-11

**Authors:** Kun Lin, Yuri Zarevich Totoev, Hongjun Liu, Tianyou Guo

**Affiliations:** 1 Shenzhen Engineering Lab for Wind Environment and Technology, Shenzhen Key Lab of Urban & Civil Engineering Disaster Prevention & Reduction, Shenzhen Graduate School, Harbin Institute of Technology, Shenzhen 518055, China; linkun.hit@gmail.com (K.L.); guotianyou1992@gmail.com (T.G.); 2 Centre for Infrastructure Performance and Reliability, The University of Newcastle, University Drive, Callaghan NSW 2308, Australia; Yuri.Totoev@newcastle.edu.au

**Keywords:** infilled RC frame, dry stacked panel, semi-interlocking masonry, cyclic test, failure mode, stiffness, energy dissipation, parallel model, mechanism

## Abstract

In order to improve the energy dissipation of the masonry infilled frame structure while decreasing the stiffening and strengthening effects of the infill panels, a new dry stacked panel (DSP) semi-interlocking masonry (SIM) infill panel has been developed. In this paper, the material properties of DSP and a traditional unreinforced masonry (URM) panel have been evaluated experimentally. A series of cyclic tests were performed to investigate the cyclic behaviour of the reinforcement concrete (RC) frame with different infill panels. The failure modes, damage evolution, hysteretic behaviour, stiffness degradation and energy dissipation were compared and analysed. We concluded that DSP is capable of significantly improving the seismic energy dissipation due to its hysteretic behaviour when the frame is in elastic stage without increasing the stiffness of the frame. Therefore, DSP or SIM panels can be considered as frictional dampers. Based on the experimental results, the influence of DSP was examined. Using the parallel model, the hysteretic loops of DSP subjected to different load cases were achieved. The typical full hysteretic loop for DSP could be divided into three distinct stages of behaviour: packing stage, constant friction stage and equivalent strut stage. The connection between the panel and the frame had a great effect on the transferring of different mechanical stages. The constant friction stage was verified to provide substantial energy dissipation and benefits to the ductility of the structure, which, therefore, is suggested to be prolonged in reality.

## 1. Introduction

Although masonry is one of the oldest and most popular building materials, it has limited use in seismic areas because of the low tensile strength and brittle behaviour of modern slender traditional unreinforced masonry (URM). To achieve better seismic performance, masonry has been combined with other stronger materials. One such dual structural system is the reinforcement concrete (RC) frame structure with masonry infill panels. These infilled frames are very common all around the world because they are practical and economical. However, their seismic performance remains questionable, especially in high seismic areas.

Masonry infill panels are usually designed as non-structural elements, whose only role is to shelter from the elements and to divide space. However, in reality, panels almost unavoidably interact with the frame during strong earthquakes, which makes the realistic structural response different from the designed one, therefore often resulting in a serious damage [1,2,3].

Previous research shows that the confining effect of the frame could improve the seismic behaviour of traditional URM panels [4,5,6]. However, serious damage was generally found both in the RC frame and masonry panels after an earthquake, which made the structures unable to continue to be used. Furthermore, infill panels are generally not distributed evenly through the structure, therefore increasing the complexity of the seismic response and sometimes resulting in the soft story effect.

Adding dampers to the frame could improve its energy dissipation [7,8,9] and seismic behaviour. However, the requirement of additional constructional cost made it difficult to popularize. As masonry panels are often an integral part of the frame structure, using them to increase the energy dissipation without significantly affecting the overall structural stiffness could be of great benefit to the seismic design.

According to previous research, the uncertainty in the characteristics of the masonry infills has great impact on the infilled RC frame’s performance, especially on its limit states of damage limitation [10,11,12]. Furthermore, the influence of the mortar joint on the seismic behaviour of the masonry infilled frame is significant [13,14]. If no mortar joint exist, dry stack masonry (DSM) is expected to exhibit a more stable performance. Actually, the dry stacking method was firstly used almost five thousand years ago; the Pyramids in Egypt are some of the most famous examples [15]. In this kind of masonry, bricks are assembled without mortar; therefore, it is also mortar-less masonry [16,17]. Because it is easy to build and is demountable, therefore, reducing the energy used in building, DSM will achieving higher material efficiency and lower costs. Because of its advantages, dry stack masonry is still popular in buildings around the world, especially in developing counties [18,19,20]. Previous research of dry stack masonry focused on the lateral capacity and the seismic behaviour of the unreinforced dry stack masonry [21]. Results show that the failure criteria of dry stack stone can be considered as a Mohr-Coulomb failure [22,23,24], and the strength of dry stack units does not make a significant difference in the resistance to lateral loads [25,26]; the interlocking and friction between units govern the lateral load-bearing capacity, and the type of wall boundary conditions and the vertical compression level were confirmed as two important factors for the failure mode [25,27]. Although considerable nonlinear deformations have been attained [21,22], un-framed dry stack masonry walls exhibited little energy dissipation because of the rocking failure mechanism.

**Figure 1:**
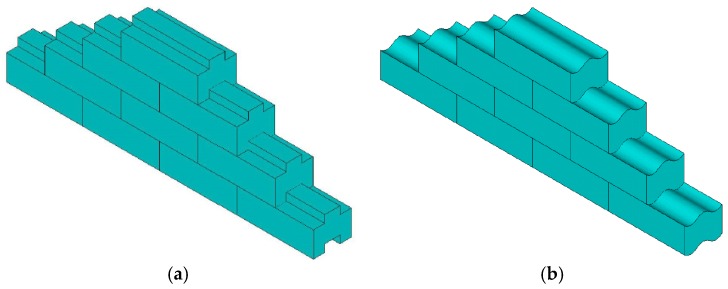
Semi-interlocking masonry units: (**a**) Rectangular interlocking; (**b**) Circular interlocking.

Out-of-plane behaviour is one major factor of restricting the application of DSM [28,29]. According to the authors’ previous research [17], semi-interlocking units have been used to build the dry stack panel. The semi-interlocking masonry panel allows relative sliding in-plane of the panel and maintains out-of-plane stability, as shown in Figure 1. The dry stack panel (DSP) no longer remains a non-structural element; instead, it should be considered as a “damper”, which contributes primarily to the energy dissipation during earthquakes.

This paper describes cyclic tests on the RC frame with different infill forms (not infilled, traditional unreinforced masonry panel (TMP) infilled and DSP infilled) of the panels and investigates the panel response mechanisms and its contribution to the entire structural energy dissipation. Series hysteretic loops have been achieved to analyse the frictional behaviour and mechanism of DSP.

## 2. Experimental Program

In order to investigate the in-plane cyclic behaviour of frames infilled with SIM panels, the testing program was carried out on a full-scale RC frame with a dry stacked masonry panel (DSP). In this initial phase of research, only the in-plane behaviour of frames was considered; no out-of-plane loads were applied; no interlocking against out-of-plane displacements of bricks was required; and none were used. Hence, DSP in these tests is considered to represent prototype SIM panel. Testing was done in three stages:

(1) The first cyclic displacement test was performed on a bare RC frame. Displacements were limited to avoid plastic strains in the frame.

(2) DSP was built inside the frame using solid concrete bricks, and the second cyclic displacement test was performed on the frame infilled with DSP. Displacements were limited to minimize plastic strains in the reinforcement. After this, the DSP was disassembled, and the cyclic test at the highest displacement level achieved in the first bare frame test was repeated to evaluate the damage to the frame during the second test.

(3) A traditional unreinforced masonry panel (TMP) was built inside the frame using the same bricks, and the third cyclic displacement test was performed. Displacements in the third test were increased until destruction of the specimen.

### 2.1. Specimen Description

**Figure 2:**
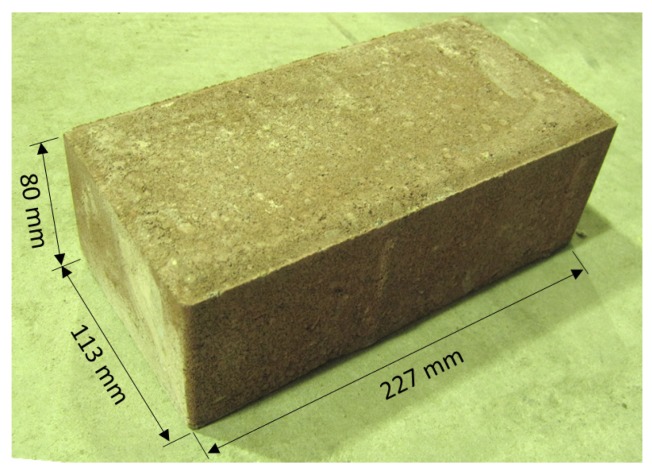
The dimensions of solid concrete bricks.

Solid concrete bricks shown in Figure 2 with dimensions of 227 mm × 113 mm × 80 mm were used for both dry stack panels (DSP) and traditional masonry panels (TMP).

**Figure 3:**
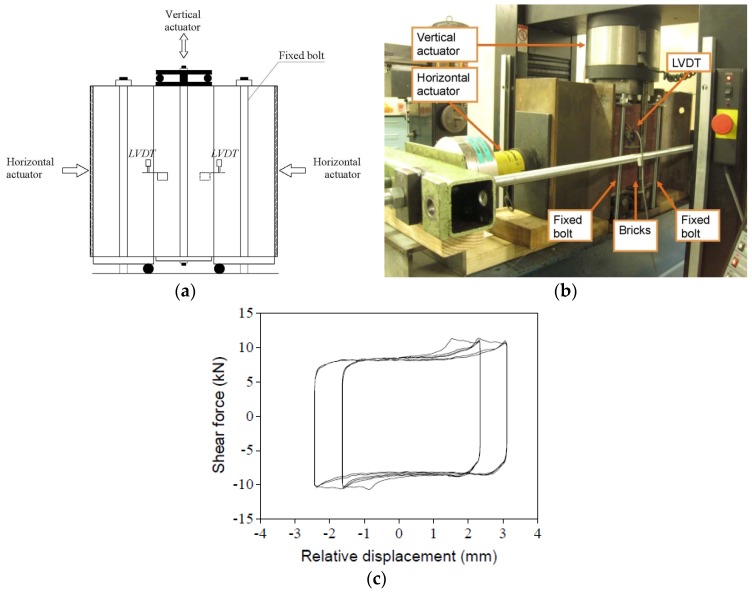
Compression-shear test of dry stacked masonry triplets: (**a**) Schematic diagram of the test; (**b**) Test setup; (**c**) Typical test result. LVDT, linear variable displacement transducer.

Material tests on brick and mortar include standards tests such as the compressive strength tests on a single brick [30], the lateral modulus of rupture test [30], the bond wrench test [31] the and compressive strength test on both dry stacked and traditional masonry prisms [31], as well as the friction test on dry stacked masonry triplets. The mechanical properties of the bricks and mortar are summarized in Table 1. The cyclic triplet test was a variation of the European standard triplet shear test [32]. The test setup is shown in Figure 3a,b. In this test, two independent actuators have been used: one for the vertical cyclic displacement; the other for the horizontal constant pressure. The real-time vertical shear load was recorded from the embedded pressure sensor in the vertical actuator; the relative displacement was recorded from the linear variable displacement transducers (LVDTs).

Three levels of compression were used for the triplet test: 0.1, 0.3 and 0.5 MPa. For each pressure case, 3 specimens were tested. For each specimen under each pressure level, 4 cyclic displacements-loadings were applied; these are: ±0.8, ±1.6, ±2.4 and ±3.2 mm; the corresponding velocities are: 1 mm/min, 2 mm/min, 2 mm/min and 4 mm/min. For the dry stack prism, the shear and the normal stress comply with the Mohr-Coulomb criterion; a frictional factor of 0.66 with a correlation coefficient of 0.99 was determined. The typical hysteretic loop is shown in Figure 3c. Detailed test results can be found in the literature [33].

**Figure 4:**
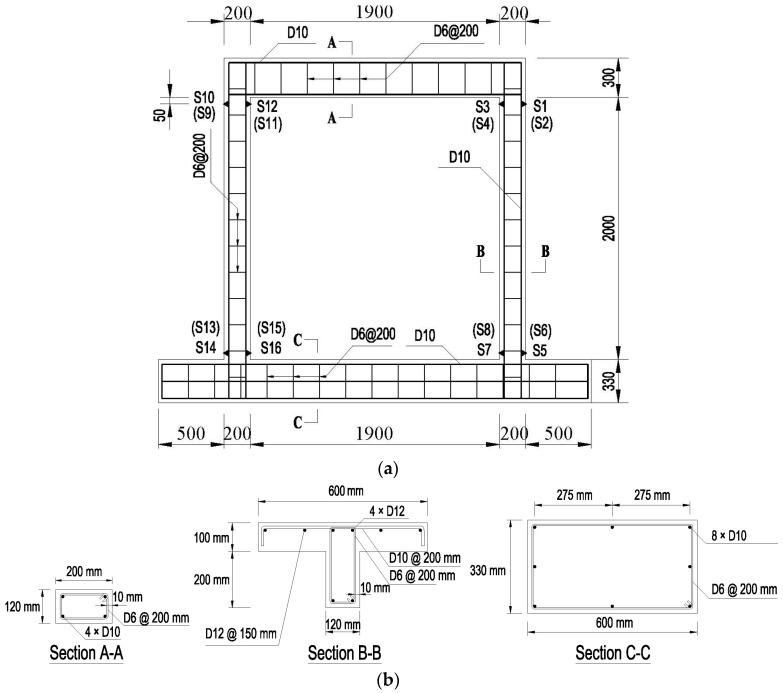
Frame details: (**a**) Instrumentation of frame (unit: mm); (**b**) Section details. S, strain gage.

The RC frame dimensions, reinforcement details and location of the strain gages are shown in Figure 4. Columns were 120 mm × 200 mm; the base beam, 330 mm × 600 mm; and a slab, 100 mm × 600 mm, was cast as a part of the top beam. The mechsanical properties of the frame elements were tested and are summarized in Table 1. The yield strengths of the reinforcement (D12: *f*_y_ = 400 MPa; D10: *f*_y_ = 400 MPa; D6: *f*_y_ = 210 MPa) were also determined in a preliminary test, where D12, D10, D6 means the reinforcement bar with a diameter of 12 mm, 10 mm and 6 mm respectively.

There were 25 layers and 22 layers in the dry stack panel and traditional masonry panel, respectively. During construction of the dry stack panel, bricks were cut to achieve a tight fit between columns; the top layer was cut to fill the frame. Even though, it was difficult to achieve perfect contact between the panel and the top beam. There was an uneven approximately 1-mm gap left between them. A similar, but wider (approximately 60 mm) gap was formed during construction of the traditional masonry panel, which was filled with dental plaster. The 1:1:6 (cement:lime:sand by volume) mortar was used for the traditional masonry panel; the thickness of mortar joints was around 8 mm. A small amount of Bycol (a common Australian air entrainer, Recochem Inc., Sydney, Australia) was added to improve the workability of the mortar.

### 2.2. Cyclic Tests

**Figure 5:**
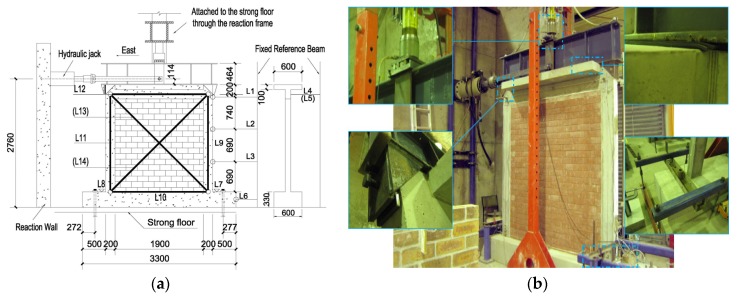
Experimental setup: (**a**) Test setup (unit: mm); (**b**) Photos. L, LVDT.

Based on previous research [34], a series of cyclic tests was set by the authors, shown as Figure 5a. The frame was bolted to the strong floor. The vertical load was applied by the hydraulic jack through the steel upper spreader beam. A thin neoprene plate was placed between the frame and upper spreader beam to ensure uniform load distribution over the specimen. The horizontal load was applied by a hydraulic actuator, which was fixed to the reaction wall. The installation photo is shown in Figure 5b.

Two types of instruments were employed during the tests: electrical strain gages and linear variable displacement transducers (LVDTs). The applied load and displacement of the hydraulic actuators were also measured. The position of LVDTs (abbreviated as “L”) and strain gages (abbreviated as “S”) are shown in Figure 4a and Figure 5a (brackets mean the LVDT was located on the opposite side).

**Figure 6:**
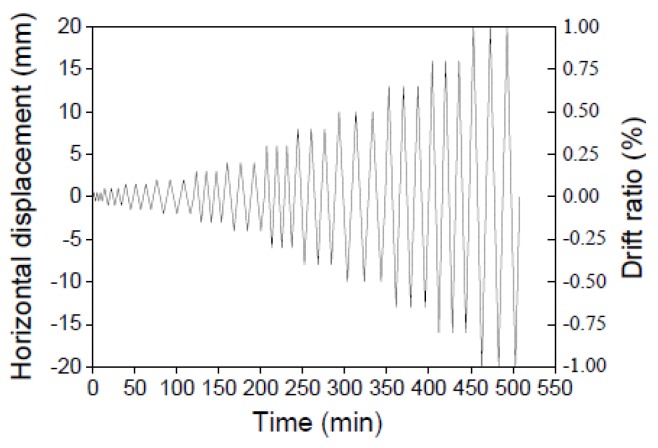
Displacement history.

To simulate the gravity load, a constant compressive stress equal to 0.3 MPa, which is considered typical for a 3-story residential building, was applied during the test. The specimen was loaded in-plane according to the displacement history shown in Figure 6. The eastbound force and the displacements were considered “positive”. The displacement rates are listed in Table 2.

In order to avoid plastic damage to the RC frame, the actuator travel was limited both in the bare RC frame and the dry stack panel test. The maximum travel of 10 mm was applied in the bare frame test, and the maximum travel of 16 mm was applied in the dry stack panel test. The maximum travel of 20 mm was applied in the traditional masonry panel test. Cracks were marked during cycling as they appeared. After each cycle, the loading was stopped, and the crack pattern was photographed.

The applied horizontal load and the horizontal displacement at the top of the frame (measured by LVDT1) were used to evaluate the cyclic behaviour and the energy dissipation in the structure. In this way, the contamination of results potentially caused by the slip between the spread beam and the frame and by the free play in the experimental setup can be effectively avoided. The story drift was calculated by dividing the measured displacement of LVDT1 by the panel height of 2 m.

## 3. Experimental Results

### 3.1. Crack Patterns

As the applied displacement was limited, there almost no visual damage occurred during the first bare frame test. Only when the amplitude of cycles reached a maximum of approximately 9 mm, there was a small hairline coverable crack observed at the left top corner of the frame.

**Figure 7:**
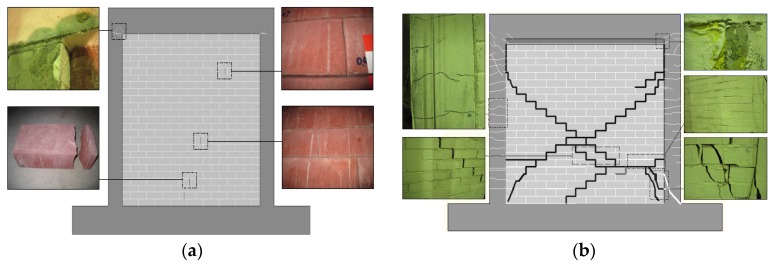
Final crack patterns: (**a**) Reinforcement concrete frame with the dry stacked panel (DSP); (**b**) RC frame with the traditional masonry panel (TMP).

There were some small coverable cracks observed in the test with DSP at both top corners of the frame. The first crack was observed at the same location and the same amplitude as in the bare frame test. The second crack was observed in the opposite top corner at the next level of displacements of approximately 11 mm. Four bricks cracked during the test, as shown in Figure 7a. However, the distribution of cracked bricks appeared to be random. This cracking could be attributed to the pre-existing damage to those bricks.

A typical diagonal cracking failure was achieved in the test with TMP. The crack was found between the top of the left column and the panel at the story drift of 0.063%. The first crack in the masonry panel was found at the bottom right corner at the story drift of 0.099%. The origin of this crack was six bricks from the bottom, and it stepped down through the mortar joints all the way to the middle of the bottom edge of the panel. No new cracks were observed in the panel until the story drift of 0.78%. Cracks in the columns began to develop at the story drift of 0.034%, and their number and widths kept increasing through the test.

The specimen failed during the second cycle at the story drift of 0.78%. The failure was sudden, and about a 15 mm-wide diagonal crack opened immediately. At the same time, the right column developed a major crack at the bottom. The final crack patterns are shown in Figure 7b.

### 3.2. Hysteretic Behaviour

**Figure 8:**
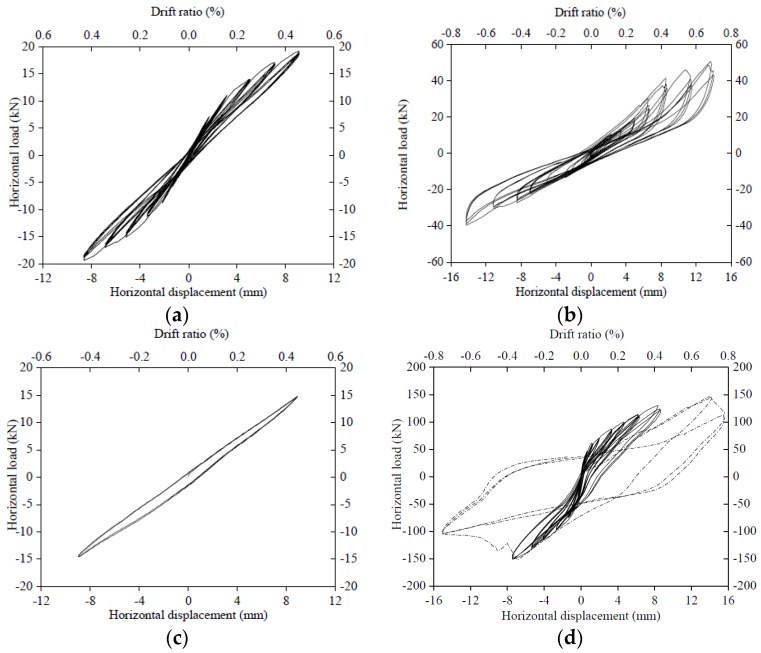
Hysteretic loop of the frame with different infilled forms: (**a**) Bare frame; (**b**) RC frame with DSP; (**c**) Double check test on the bare frame; (**d**) RC frame with TMP.

Plots of the in-plane story drift *versus* the in-plane lateral force for all cyclic tests are shown in Figure 8.

In the first bare frame test, the hysteretic loops closely repeated each other for displacement cycles of the same amplitude. The loops were also symmetric at all levels of the displacement. In the test with DSP, the hysteretic loops were stable, but not symmetrical. The maximum force recorded in the “negative” direction was lower than the “positive” force at the same displacement. This asymmetric behaviour can be attributed to the existence of the uneven gap between the frame and the top of the masonry panel, as mentioned above. The “positive” loops have a large area, which is indicative of greater energy dissipation. They are assumed to better represent the cyclic behaviour of a dry stack panel without the gap at the top.

In the test with TMP, the observed behaviour was typical for a framed unreinforced masonry panel subjected to cyclic loading. After an initial elastic stage, loops exhibited static hysteresis attributed to plastic damage in the structure. There was no signal crack in the panel as in some confined masonry cyclic tests. The maximum strength was reached at drift levels close to 0.5%. At this peak, an ideal diagonal crack suddenly opened across the masonry panel. After the peak load was reached, the story drift kept increasing to 0.8% with the strength reduced by 30% from the peak value (dotted line in Figure 8d).

### 3.3. Lateral Stiffness

Stiffness degradation is assessed using secant stiffness of the peak-peak value determined from each complete hysteresis loop. In order to analyse the effects of the panel on frame stiffness, the relative stiffness *K′* was used, which was calculated by Equation (1).

(1) $$K^{\prime }=\frac{K_{i}}{K_{0}}$$

where *K_i_* is the average secant stiffness of each story drift; *K*_0_ is the initial secant stiffness of bare frame.

The energy dissipation capacity is an important parameter in the evaluation of the potential seismic resistance of the structure. It was calculated for all three tests as the area inside the hysteretic loops.

The story drift (α), relative stiffness (*K′*) and energy dissipation of three cycles (*W*_D_; this will be discussed in next section) are listed in Table 3. Comparing the contribution of DSP and TMP to the overall structural response, the following observations can be made:

(1) The DSP increased the initial stiffness of the assembly slightly to approximately two-times that of the bare frame. The TMP increased the initial stiffness of the assembly massively to approximately 30-times that of the bare frame;

(2) In the elastic response stage of the frame (α ≤ 0.18%) [35], the secant stiffness of the frame with DSP is almost the same as bare frames, while the corresponding value for the test with TMP is about six-times that.

### 3.4. Energy Dissipation

The energy dissipation in the test with DSP was caused mainly by the relative slip and friction between bricks inside the panel. There are two distinct stages of the response with two different mechanisms of energy dissipation. The story drift of approximately 0.2% was a threshold. Before that, the energy dissipation in the frame was due to the internal thermal friction in the elastic stage of response and some micro-structural damage in the plastic stage. The energy dissipation in the panel was due to the constant friction between bricks caused by panel self-weight only. After the gap between the frame and panel was closed, the energy dissipation was increasing partially because more micro-structural cracks were developing in the frame, but mainly because compression and, as a result, friction forces were increasing between bricks.

The energy dissipation in the traditional masonry test was caused mainly by the macro-structural damage to mortar joints, concrete frame and bricks. The first visible cracking of the frame was observed at the story drift of 0.06%. Before that, energy dissipation was mainly elastic or caused by micro-structural cracking.

The total energy dissipation in the test with TMP before the onset of cracking in the frame at the story drift of 0.06% was 91.87 kN·mm. In the test with the dry stack panel, the new cracking in the frame was observed at the story drift of 0.7%. The total energy dissipation before that was 1568.2 kN·mm, which is approximately 17-times higher than in the traditional masonry test.

The maximum horizontal load for the bare frame was 18.05 kN. The total energy dissipation before reaching this load was 91.812 kN·mm. At the same load level, the total energy dissipation in the traditional masonry test was 3.07 kN·mm. In the dry stack panel test, the total energy dissipation before reaching this load level was 147.32 kN·mm, which is almost 50-times higher than in the traditional masonry test.

### 3.5. Response Mechanisms

The response of this type of dual structural system depends on the combined action of both the RC frame and dry stack masonry panel. As a classical type of structure, the lateral capacity of the RC frame has been comprehensively researched. The dry stack masonry behaviour attracted less research attention. The interaction between the two is not yet fully understood. It appears that the DSP exhibits Mohr–Coulomb behaviour. However, the combined action of the frame and the panel is not a simple superposition of two mechanisms; the gap between the top of the panel and the frame plays a key role in the frame-panel interaction.

**Figure 9:**
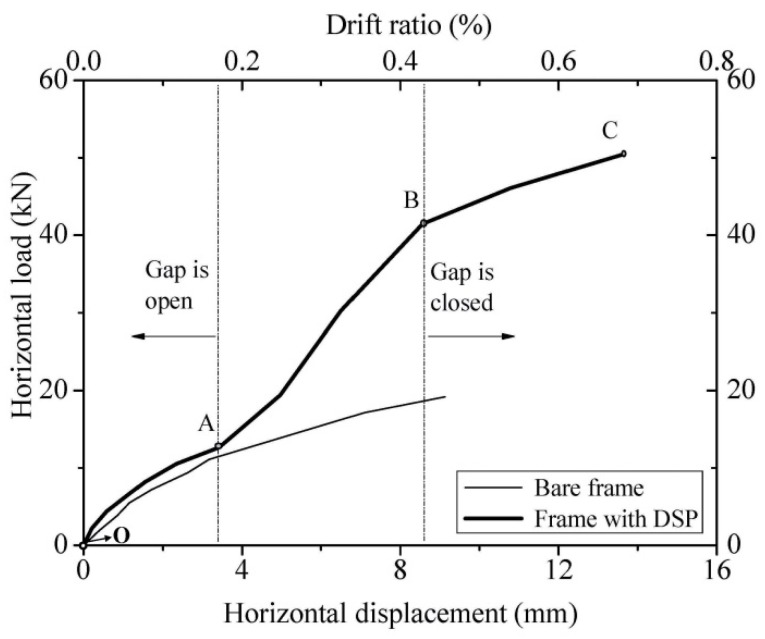
Envelope curves.

**Figure 10:**
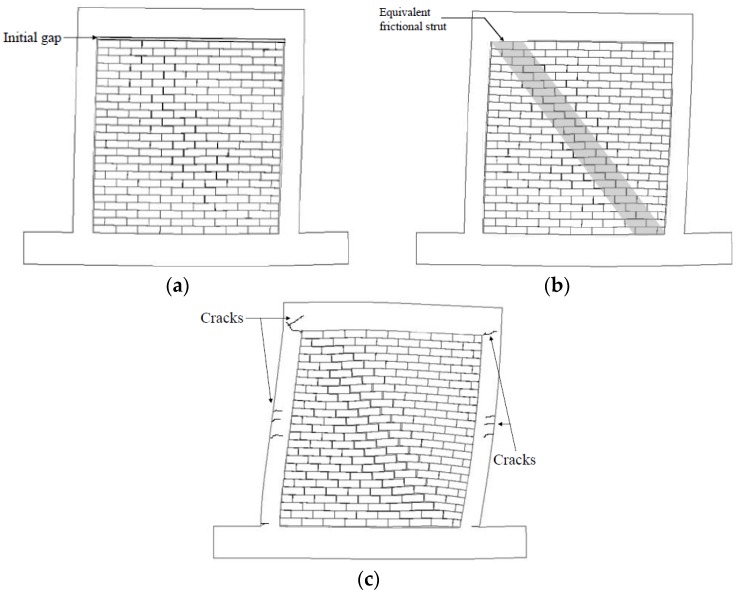
Response mechanisms: (**a**) Constant friction stage; (**b**) Equivalent frictional strut stage; (**c**) Plastic stage.

The envelope response curve for the structure was attained from Figure 8 and shown as Figure 9. Compared to the envelope curve for the bare frame, the structural response can be divided into three stages: OA (0% to ~0.2% storey drift); AB (~0.2% to ~0.4%); BC (beyond ~0.4%). Three corresponding response mechanisms are shown in Figure 10.

First stage (OA), constant friction stage: The RC frame is interacting with the dry stack panel compressed by its own weight only. The frame is not in contact with the top of the panel, because the gap between them is open. Frictional forces between bricks are therefore relatively small and constant.

Second stage (AB), equivalent frictional strut stage: The RC frame is now in contact with the top of the panel. This has two significant effects: (i) the friction between bricks is increasing due to increasing compression of the panel by the frame; and (ii) a type of compressive strut is formed within the panel similar to the traditional in-filled masonry panel. Compared to the first stage (OA), the stiffness of the structure has increased.

Third stage (BC), plastic stage: One of the components of the dual structural system exceeds its yield limit and is cracking. In this case, the RC frame is cracking and exhibits plastic behaviour. Compared to the second stage (AB), the stiffness of the assembly has decreased to about the same as in the first stage (OA). The observed damage of the RC frame is assumed to be the reason. At this stage, the cracks developed at column/beam connections, as shown in Figure 7a. This stage cannot be compared to bare frame experimental results only because the bare frame test had been stopped at the 10-mm jack travel to avoid damaging the frame.

## 4. Evaluation of Dry Stacked Panel (DSP) Behaviour

### 4.1. Evaluation Method

Comparing the difference between the bare frame and the frame with DSP, we can see that the contribution of DSP to the combined structural response is significant.

According to the series of tests and theoretical analysis, five different failure modes of RC frames with traditional infill panels have been identified [36,37]. DSP infills behave quite differently compared to TMP. However, traditional infill panels with weak mortar framed by a strong RC frame are in some respects similar to DSP. Those TMPs are prone to diagonal/sliding shear failure of the infill and the shear failure of the windward column. In this kind of dual structure, the frame and the infill panel are often considered as two parallel systems with displacement compatibility at the compression corners. This approach is called the “parallel model”.

**Figure 11:**
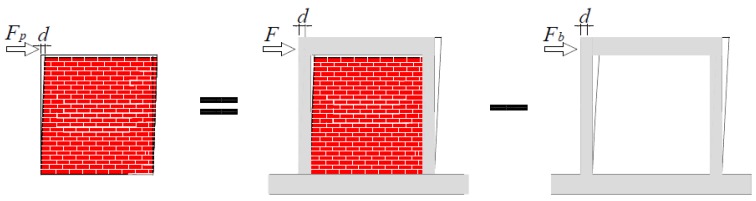
Parallel model approach.

In the DSP structure, there is no bonding between bricks, and the connection is weak; therefore, the “parallel model” was adopted for the analysis of lateral bearing capacity. The bearing load on the structure is resisted jointly by the frame and the panel, as expressed in Equation (2).

(2) $$F=F_{b}+F_{p}$$

where *F*, *F_b_* and *F_p_* are the lateral capacity of the whole structure, the bare frame and the DSP, respectively. The bearing load on the panel could be attained by subtracting the bearing load on the frame only from the bearing load on the frame/panel assembly at the same horizontal displacement, as shown in Figure 11.

Strictly speaking, the principle of Equation (2) is applicable only as long as the behaviour of the frame remains linear. This evaluation based on the parallel model is extended here beyond the linear range of the frame resistance only to gain insight into energy dissipation in the DSP. For this analysis, the hysteretic loops have been processed in the following three steps:

(1) Discretization step: The two groups of curves (bare frame and frame with DSP; see Figure 8a,b) were discretised using displacement increments of 0.001 mm with linear interpolation between these points to ensure that the shape of the curve is not distorted.

(2) Regularization step: The bare frame curve was scaled up or down to match its peak displacement value to that of the frame with the DSP curve. The original and regularized data are listed in Table 4, where *D*, *F* is the horizontal displacement and corresponding lateral force achieved from DSP infilled frame experiment; *d*_B_ and *F*_B_ is the horizontal displacement and corresponding lateral force achieved from bare frame experiment.

(3) Decoupling step: Subtracting the bare frame data from the frame with DSP data attains the hysteretic loops of DSP.

**Figure 12:**
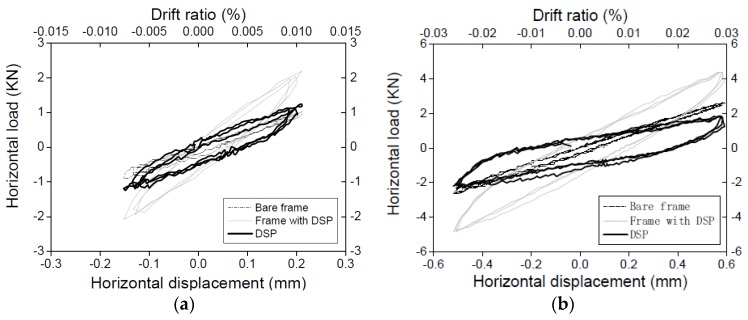

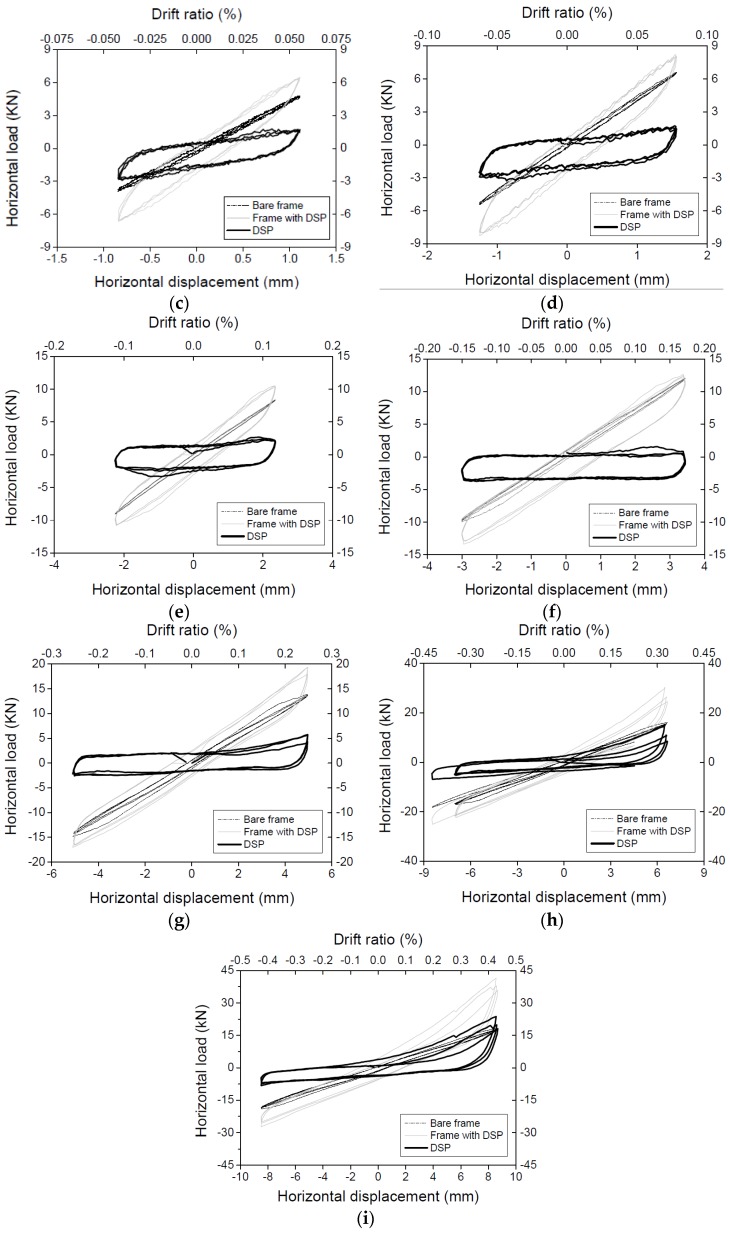
Hysteretic loops for dry stacked panel at different levels of cycling displacement: (**a**) Load Case 1; (**b**) Load Case 2; (**c**) Load Case 3; (**d**) Load Case 4; (**e**) Load Case 5; (**f**) Load Case 6; (**g**) Load Case 7; (**h**) Load Case 8; (**i**) Load Case 9.

The frame behaviour during the constant friction and the equivalent friction strut stages of response described above is almost linear elastic. However, some degradation of stiffness of the structure was observed between cycles at the two highest levels of displacement. This significantly nonlinear behaviour of the frame is considered unsuitable for analysis based on the accepted parallel model. Hence, the data for those two cycles was not used in the analysis. The achieved hysteretic loops for DSP are shown in Figure 12.

### 4.2. Simplified Mechanical Model

**Figure 13:**
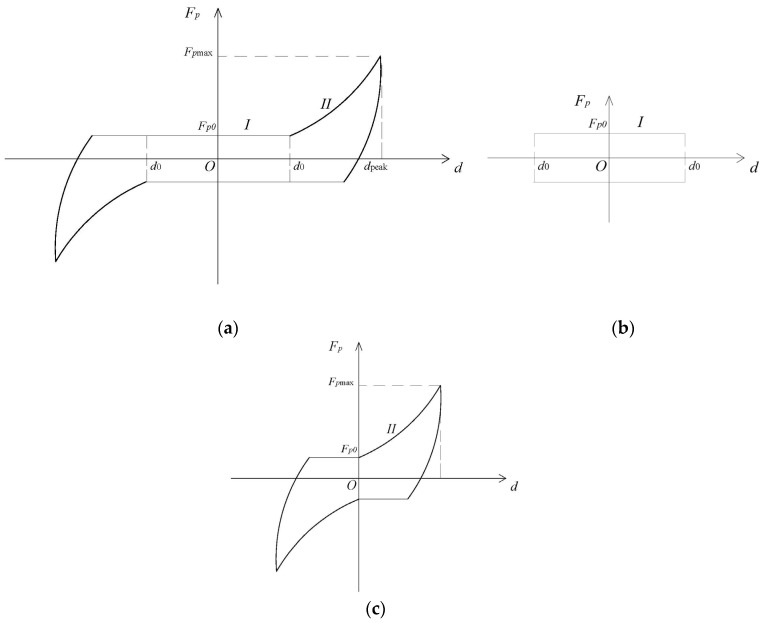
Modelling of a typical hysteretic loop for dry stack infill panel: (**a**) Full hysteretic loop for DSP; (**b**) Constant friction stage; (**c**) Equivalent strut stage

It is apparent that the hysteretic behaviour of DSP changes as the displacement increase. The typical full hysteretic loop for DSP can be assumed as in Figure 13a. There are three distinct stages of behaviour:

(1) Packing stage: At the low level of the storey drift (α < 0.03%), the induced horizontal shear forces in the panel are generally lower than the static friction forces between bricks. Hence, there is little relative sliding between bricks; just to pack bricks tightly and reach the full static friction potential. The DSP slightly increases the stiffness of the assembly, as can be seen in Load Cases 1 and 2.

(2) Constant friction stage (CF stage): With the increase of story drift (0.03% < α < 0.2%), bricks start sliding, and the relative displacement gradually increases. DSP shows typical frictional damper behaviour, which is speed independent and has the peak-peak value, which depends on the self-weight of the panel and is almost constant at about 4 kN. During this stage, the DSP acts as an effective frictional energy dissipation device (see Load Cases 3 to 6); the behaviour of DSP in the CF stage was decided by the constant friction force Fp0, which depends on the self-weight of the panel.

(3) Equivalent strut stage (ES stage): As the lateral displacement increases, the gap between the top of the panel and the frame start to close (α > 0.2%), the compression in the DSP increases with a consequent increase in the friction between bricks. This point signifies the formation of the equivalent compressive/frictional strut in the DSP. In addition to the increased energy dissipation in the panel at this stage, the equivalent strut significantly increases its stiffness contribution, as can be seen in Load Cases 7 to 9. The asymmetry of the hysteretic curves in the ES stage of our test was caused by the uneven gap between the top of the panel and the RC frame. As the gap is uneven, the hysteretic loops show asymm etric behaviour about the vertical axis.

## 5. Conclusions

A new masonry system designed to improve the seismic behaviour of RC frame with masonry panels has been developed. In this system, a dry stack panel is built with masonry units capable of sliding in-plane of a panel. A series of material and cyclic tests was carried, and the contribution of DSP was investigated.

The cracking patterns and hysteretic behaviours of the frame with different infilled panels have been researched. Typical diagonal cracking failure was achieved in the test with TMP. Different from the frame with TMP, there are almost no cracks occurring on the frame during the elastic stage; a series of full hysteresis curves was achieved for the frame with DSP, which indicates a significant energy dissipation of the DSP.

Compared to the traditional unreinforced masonry panel (which increase the stiffness of the RC frame 30 times), the DSP increases the initial stiffness of frame about two times. During the elastic response stage (α ≤ 0.18%), the secant stiffness of the DSP frame is almost the same as that of the bare frame, while this value is around six-times higher for traditional masonry panels.

The addition of DSP to a light RC frame could increase the structural energy dissipation according to the experimental results. The semi-interlocking masonry panels could be good energy dissipation devices for frame structures in seismic regions. Their influence requires further experimental research.

The typical hysteretic loops of DSP were achieved according to the parallel model based on the experimental results. The hysteretic loop of DSP exhibits typical frictional behaviour under low horizontal drift, which indicates that the DSP has the potential to be designed as a frictional damper in a reality program. The mechanism of the DSP frame has been investigated, and two distinct stages of the response have been identified: they are the constant friction response and the equivalent frictional strut response. The gap between the frame and the panel was found to have significant influence on the composite response of the structure.

## Figures and Tables

**Table 1:** Mechanical properties of the specimens.

Specimen	Density (kg/m^3^)	Elastic Modulus (MPa)	Compressive Strength (MPa)	Tensile Strength (MPa)
Bricks	2250	26,365	28.55	3.25
Traditional masonry	-	20,407	21.5	0.17
Dry stack masonry	2250	7702.3	18.3	-
Base beam	2370	32,000	21.8	2.93
Columns	2280	25,000	23.6	2.34
Top beam and slab	2350	33,000	24.1	3.30

**Table 2:** Displacement rates.

**Travel (mm)**	0.5	1	1.5	2	3	4	6	8	10	13	16	20
**Travel speed (mm/min)**	0.5	0.5	0.5	0.5	1	1	2	2	2	3	4	4
**Period (min)**	4	8	12	16	12	16	12	16	20	17	20	20

**Table 3:** Stiffness and energy dissipation of the RC frame with different infilled forms.

Bare Frame Test				Test with DSP				Test with TMP			
α (%)	*F* (kN)	*K′*	*W*_D_ (kN·mm)	α (%)	*F* (kN)	*K′*	*W*_D_ (kN·mm)	α (%)	*F* (kN)	*K′*	*W*_D_ (kN·mm)
0.02	1.90	1.00	0.51	0.01	2.19	2.44	0.65	0.001	5.18	30.03	0.47
0.04	3.80	0.93	1.25	0.03	4.39	1.68	5.26	0.01	18.33	27.48	3.07
0.06	5.50	0.88	2.23	0.06	6.5	1.34	12.85	0.02	36.36	21.06	7.32
0.09	7.16	0.85	3.31	0.08	8.21	1.16	22.66	0.03	47	17.6	15.38
0.13	9.40	0.75	6.47	0.12	10.54	0.92	51.03	0.06	61.61	11.25	65.62
0.16	11.08	0.67	15.15	0.17	12.65	0.79	75.53	0.1	70.63	8.62	121.47
0.25	14.03	0.55	38.31	0.25	19.38	0.71	147.32	0.17	87.26	5.94	313.61
0.36	17.13	0.48	64.68	0.33	30.28	0.72	255.97	0.24	99.92	4.75	454.85
0.46	19.19	0.42	91.81	0.44	41.46	0.75	441.62	0.32	113.9	4.09	657.05
—	—	—	—	0.58	46.10	0.64	555.30	0.43	129.98	3.40	1381
—	—	—	—	0.70	50.50	0.61	758.87	0.55	135.27	2.83	2594
—	—	—	—	—	—	—	—	0.78	145.99	1.44	6933

**Table 4:** Calculation of the force contribution of DSP.

Load Cases	Frame with DSP		Bare Frame				Force Contribution of DSP	
			Original Data		Regularized Data			
	*D* (mm)	*F* (kN)	*d*_B_ (mm)	*F*_B_ (kN)	*d*_B_′ (mm)	*F*_B_′ (kN)	Absolute Value (kN)	Average Value (kN)
1	0.21	2.19	0.39	1.90	0.21	1.02	1.17	1.15
	−0.15	−2.08	−0.17	−1.10	−0.15	−0.96	1.12	
2	0.58	4.39	0.84	3.80	0.58	2.61	1.78	1.97
	−0.51	−4.86	−0.47	−2.50	−0.51	−2.69	2.17	
3	1.11	6.50	1.27	5.50	1.11	4.84	1.66	2.17
	−0.84	−6.64	−0.87	−4.10	−0.84	−3.96	2.68	
4	1.55	8.21	1.70	7.16	1.55	6.53	1.68	2.26
	−1.24	−8.24	−1.31	−5.75	−1.24	−5.41	2.83	
5	2.34	10.54	2.63	9.40	2.34	8.38	2.16	2.01
	−2.23	−10.85	−2.19	−8.81	−2.23	−9.00	1.85	
6	3.43	12.65	3.17	11.08	3.43	11.99	0.66	2.04
	−3.00	−13.38	−3.42	−11.36	−3.00	−9.96	3.42	
7	4.97	19.38	5.07	14.03	4.97	13.75	5.63	3.90
	−5.10	−17.03	−5.19	−15.13	−5.10	−14.86	2.17	
8	6.50	30.28	7.09	17.13	6.50	15.70	14.58	9.97
	−6.92	−22.45	−6.90	−17.04	−6.92	−17.10	5.35	
9	8.56	41.46	9.13	19.19	8.56	17.99	23.47	15.86
	−8.49	−27.27	−8.65	−19.38	−8.49	−19.02	8.25	
